# Adolescent disordered eating and epigenetic age acceleration

**DOI:** 10.1038/s41380-026-03595-3

**Published:** 2026-04-10

**Authors:** Madeleine Curtis, David Balfour, Phillip Melton, Ashleigh Lin, Zoe Kleinig, Sarah Cohen-Woods

**Affiliations:** 1 Blackbird Initiative, Flinders University Institute for Mental Health and Wellbeing, Flinders University, Bedford Park, South Australia Australia; 2 Menzies Institute for Medical Research, University of Tasmania, Hobart, Tasmania Australia; 3 School of Global and Population Health, University of Western Australia, Crawley, Western Australia Australia; 4 The Australian Research Centre in Sex, Health and Society, La Trobe University, Melbourne, Australia; 5 Flinders Centre for Innovation in Cancer, College of Medicine and Public Health, Flinders University, Adelaide, South Australia Australia

**Keywords:** Psychology, Genetics, Psychiatric disorders

## Abstract

Disordered eating is associated with a range of poor health outcomes that affect various body systems, including immune, metabolic, and stress-related processes. Biological ageing represents a potential mechanism through which disordered eating may impact these processes and can be measured through epigenetic age acceleration. Epigenetic age acceleration has been explored in various psychiatric disorders, but no study has investigated epigenetic age acceleration in those with eating disorders, or disordered eating. The present study examined the association between disordered eating at ages 14 and 17, and epigenetic age acceleration at age 17 in a representative community cohort (*N* = 797). Disordered eating was assessed using global scores on the Eating Disorder Examination Questionnaire (EDE-Q), as well as scores on behavioural items relating to restriction, bingeing, and purging. After FDR correction, restriction at age 14 was longitudinally associated with a small (β = 0.09) increase in DunedinPACE, but not PhenoAge, GrimAge, Horvath, or Hannum. The association between age 14 restriction and DunedinPACE was partially mediated through higher BMI at ages 14 and 17, consistent with a role for BMI in the adverse health outcomes associated with disordered eating. No concurrent relationships were found between disordered eating at age 17 and epigenetic age acceleration. Findings suggest that third generation clocks may capture some of the long-term biological correlates of disordered eating, but – because the effect was small – it may not be clinically relevant. Replication using large community samples is required, along with studies that measure disordered eating and epigenetic age at multiple time points, to enhance causal inference.

## Introduction

Disordered eating encompasses both clinical eating disorders, and disordered eating-related behaviours and cognitions that may not meet full diagnostic criteria for an eating disorder, but still cause significant distress. Studies have shown that disordered eating behaviours such as dietary restriction, excessive exercise, binge eating, and purging, often result in severe health issues affecting multiple organ systems, including immune, metabolic, and stress-related processes [1–3]. Further, the cognitions that accompany disordered eating behaviours, such as distorted thoughts about body weight or the need to control food intake, often cause increased psychological stress [4], leading to the activation of physiological stress responses including increased inflammation, lowered immune response, and impaired psychosocial functioning [5].

Biological ageing, referring to the progressive decline of physiological function, tissue structure, and organ integrity over time, may be a pathway through which disordered eating influences these health outcomes [6]. Biological ageing can be defined through multiple biological systems and has been measured using clinical biomarkers, such as white blood cell count and C-reactive protein [7, 8], and also epigenetic variation (epigenetic age) through the use of epigenetic clocks which measure changes in DNA methylation patterns [7, 9–11]. DNA methylation involves the addition of a methyl group to a cytosine base at a CpG site and can result in affected genes being suppressed or silenced [12, 13]. DNA methylation has been investigated in those with eating disorders, however most studies have focused on anorexia nervosa (AN) [14], with a smaller number investigating bulimia nervosa and binge eating disorder [15–22]. No studies have investigated DNA methylation in people with disordered eating more broadly.

DNA methylation studies on eating disorders using candidate gene and global methylation approaches have been mixed and yielded inconsistent findings [23]. More recently, epigenome-wide association studies (EWAS) have identified differentially methylated CpG sites associated with eating disorders (primarily AN) in genes related to metabolic, psychological, and immune functioning [19, 24–28]. Studies have consistently demonstrated increased DNA methylation among individuals with AN [24–27], and chronicity of AN has been associated with variations in DNA methylation in regions linked to genes associated with immunity, anxiety, metabolism, serotonin, insulin, and central nervous system function [25, 26, 28].

Although no studies have explicitly explored DNA methylation in people with disordered eating as a global construct, factors associated with disordered eating, including diet [29], stress [30, 31] and exercise [32], have been correlated with DNA methylation in peripheral tissues (e.g., blood). In addition, the characteristic behaviours responsible for adverse health outcomes (e.g., restriction, binge eating, self-induced vomiting) exist across the entire disordered eating spectrum, including clinical eating disorders [4]. Thus, it is likely that altered DNA methylation patterns extend to disordered eating presentations beyond clinical diagnoses. DNA methylation variations in metabolic, psychological, and immune pathways have been associated with age-related biomarkers and can be used to measure epigenetic ageing [33]. As these pathways are also implicated in the poor health outcomes associated with disordered eating, epigenetic age acceleration represents a potential mechanism connecting disordered eating and these adverse health effects.

Epigenetic age acceleration (EAA), referring to the difference between an individual’s estimated epigenetic age and their chronological age, can be measured using epigenetic clocks [33, 34]. Differences in EAA have been identified in individuals with various psychiatric disorders, however – to our knowledge – no studies have investigated EAA in individuals with eating disorders or disordered eating [35]. To address this gap, the present study examined the relationship between disordered eating and EAA in an Australian adolescent cohort (the Raine Study). Given that there is an established association between BMI and disordered eating [36], as well as an increasing body of literature supporting a relationship between BMI and DNA methylation changes [28, 37–43], we also explored the mediating role of BMI. Thus, the specific aims of this study were to 1) perform longitudinal analyses to determine whether disordered eating at age 14 predicted EAA at age 17, 2) conduct cross-sectional analyses to investigate whether disordered eating at age 17 is associated with EAA at age 17, and 3) explore whether BMI mediates the relationship between disordered eating and EAA.

## Method

### Participants and procedure

The present study uses data from the Raine Study, an ongoing longitudinal birth cohort in Western Australia. Generation 1 (Gen1) participants were recruited from King Edward Memorial Hospital and nearby private clinics in Perth, Western Australia. Those invited to participate included women who were between 16 and 20 weeks’ gestation, had sufficient proficiency in English, were expected to deliver at the hospital, and who intended to remain living in Western Australia. A total of 2968 Gen1 participants enrolled in the study. Generation 2 (Gen2) participants included 2868 babies (including 60 sets of twins and two sets of triplets) who were born from 2826 Gen1 mothers between 1989 and 1991 [44]. Follow-ups occurred at 34 weeks’ gestation (prebirth), and ages 1, 2, 3, 5, 8, 10, 14, 17, 18, 20, 22, 27, and 28 years, and data collection included clinical and sociodemographic information. Follow-ups were labelled using the average age of participants during each data collection period. Informed written consent was obtained from the primary caregiver (Gen1) on behalf of Gen2 participants at each follow-up, and from Gen2 participants when they were old enough to consent. All data collection follow-ups were approved by the Human Ethics Committee at Princess Margaret Hospital for Children, King Edward Memorial Hospital, and the University of Western Australia in Perth. Previous analyses of Raine Study participants have demonstrated that the sociodemographic characteristics of participants retained through to adolescence are representative of the wider Western Australian population [44, 45].

Participants eligible for inclusion in the present study were Gen2 participants who completed the Eating Disorder Examination Questionnaire (EDE-Q) at ages 14 and 17, and who had epigenetic data available (*N* = 797, 49.6% female). The mean age at year 14 was 14.08 (*SD* = 0.17, range 12.99 – 14.78) and 17.01 (*SD* = 0.22, range 16.28 – 18.32) at year 17. Ethical approval for the present study was obtained from the Flinders University Research Ethics Committee (HEL5527-1) and the University of Western Australia Human Research Ethics Committee (RA/4/20/5722). All methods were performed in accordance with relevant guidelines and regulations.

### DNA methylation profiling

Peripheral blood samples were provided by consenting Gen2 participants at the Gen2-17 year follow-up. DNA was extracted from whole-blood samples using a Puregene DNA isolation kit (Qiagen, Germany) based on a simple salting out technique [46]. Genomic DNA was treated with sodium bisulphate using Zymo EZ DNA Methylation-Gold kit (ZymoResearch, Irvine, California, USA, D5007). Epigenome-wide DNA methylation profiles were generated for 1192 (58 technical replicates) participants using the Illumina (San Diego, CA) Infinium HumanMethylation450 BeadChip Array (Illumina 450k Array) at the Centre for Molecular Medicine and Therapeutics, University of British Columbia. The median intraclass correlation (ICC) between all probes for the technical replicates was 0.997 and the median absolute difference between the technical replicates was 0.016, indicating highly reliable measurements.

### Quality control

Quality control was performed using R and Bioconductor packages *shinyMethyl* *[*47], *MethylAid* *[*48], and *RnBeads* *[*49]. Four participants were removed prior to analysis, following standard and widely-practiced quality control criteria. Three participants with poor probe quality were identified as outliers using principal components for *MethylAid* and *shinyMethyl* using default thresholds for the 450 K array [50]. The default thresholds for the Illumina 450 K array include median of methylated to unmethylated ratio = 10.5 [51], overall sample dependent ratio = 11.75 [52], bisulfite conversion = 12.75 [53], hybridisation control probe quality = 13.25 [54], and detection *p* value < 0.01 = 0.95. One participant was removed due to a sex misclassification identified through a chromosome discrepancy based on the average signal intensity for the participants. Sixty-five CpGs for which a common SNP disrupted the site leading to genotypic specific methylation levels were removed, as well as 11,648 sex chromosome CpGs, and 10,777 CpGs with a detection *p* > 0.05 in any sample. An additional 160 probes with bead counts of below three in more than 5% of samples were removed. Batch effects were still present after BMIQ was applied [55].

### Epigenetic age acceleration

Five epigenetic clocks were used to calculate EAA: PhenoAge [7], GrimAge [9], Horvath [11], Hannum [56], and DunedinPACE (Dunedin Pace of Aging Calculated from the Epigenome) [10]. PhenoAge is a second-generation mortality clock that estimates biological age using a DNA methylation algorithm predictive of mortality, incorporating nine clinical biomarkers related to inflammation, kidney function, liver function, metabolic health, and immune response, along with chronological age and DNA methylation data from 513 CpG sites [7]. GrimAge is a second-generation mortality clock that uses DNA methylation-based proxies of age, sex, self-reported smoking pack-years and biomarkers relating to inflammation, kidney function, cognitive function, appetite, insulin resistance, cardiovascular disease, and ageing-related disorders [9]. GrimAge provides an estimate of time-to-death, which is then transformed to a biological age estimate measured in years [9]. Horvath is the most widely-used first generation clock, estimating chronological age from DNA methylation in a wide range of tissue types [11]. Hannum is another popular first generation clock, producing an extrinsic estimate that explicitly uses information about age-related differences in cell composition in whole blood to estimate chronological age [56]. Epigenetic age estimates using PhenoAge, GrimAge, Horvath, and Hannum were calculated using Python scripts supplied by the clock developers [7, 9]. EAA was then calculated by regressing participant’s epigenetic age estimates onto their chronological age and calculating residual values. A positive value indicates accelerated biological ageing compared to chronological age, whereas a negative value indicates slower biological ageing. DunedinPACE is a third-generation epigenetic clock that measures the pace of biological ageing using a DNA methylation algorithm developed from 173 CpGs, selected based on longitudinal biomarker data across metabolic, cardiovascular, immune, hepatic, renal, dental, and pulmonary systems [10]. Values are standardised to have a mean of 1 and represent the pace that an individual is estimated to age in a year. Higher values indicate more accelerated epigenetic ageing, with values greater than 1 indicating accelerated biological ageing relative to chronological age [10].

### Disordered eating measures

Disordered eating in the Raine Study was assessed using 24 self-report items derived from the Eating Disorder Examination Questionnaire (EDE-Q) [57] and the Child Eating Disorder Examination (ChEDE) [58]. Adaptions were made to the original EDE-Q and ChEDE to assist with participant comprehension and completion of the questionnaire. Language was simplified for use with children, four non-diagnostic items were removed relating to restraint and eating, weight, and shape concerns, and response options were modified so that all items were measured on a 4-point Likert scale: 0 = “not at all”, 1 = “some of the time (once per week/a few times per month)”, 2 = “a lot of the time (a few times per week)”, and 3 = “most of the time (every day or nearly every day)”. Participants were asked to be conservative with their answers if they were unsure of their behaviour frequency [59]. At the Gen2-14 year follow-up, participants were administered the 24-item version of the EDE-Q, consisting of 18 subscale items and 6 behavioural items assessing symptoms over the previous 28 days. At the Gen2-17 year follow-up, participants received a shortened version of the EDE-Q, consisting of 17 questions (11 subscale items and 6 behavioural items) addressing the previous 14 days. Questions at age 17 corresponded to items 1-17 of the EDE-Q administered at age 14. The EDE-Q has demonstrated good internal consistency (Cronbach’s α ranging from 0.70 to 0.93) and has been shown to adequately discriminate between those with and without an eating disorder [60].

### Global EDE-Q scores

Pearson’s correlations revealed that global EDE-Q scores calculated at age 14 from the full (18-item) and shortened (11-item) versions were strongly correlated (*r* = 0.97, *p* < 0.001). To enable comparability between ages 14 and 17 in the present study, global scores for both time points were calculated based on the shortened 17-item version of the EDE-Q administered at age 17, using the 11 subscale items. The 11-item EDE-Q demonstrated excellent internal reliability at both age 14 (α = 0.95) and age 17 (α = 0.94). Global scores were calculated as the mean item score across the dietary restraint, eating concern, weight concern, and shape concern subscales. Scores ranged from 0–3, with higher scores indicating greater disordered eating symptom severity.

### Behavioural items

In addition to global EDE-Q scores, scores on items relating to specific disordered eating behaviours, including restriction, bingeing, and purging were assessed. Restriction was calculated as the highest score across two items assessing restraint and exercise. Purging was calculated as the highest score across two items assessing self-induced vomiting and laxative misuse. Bingeing was calculated by combining a measure of loss of control and portion size. All behaviours were measured continuously, with a possible score range of 0–3 for restriction and purging, and 0–6 for bingeing.

### Covariates

Variables known to also influence DNA methylation were included as covariates, including BMI, smoking status, and sex [40, 41, 61]. BMI was assessed using participants’ BMI centile (BMI-for-age) as it is considered more accurate for children than BMI (CDC, 2022). BMI centile was calculated from height and weight measurements at follow-ups. Percentiles were based on norms from the 2000 Centre for Disease Control United States growth charts and were calculated using the *childsds* package in R [62, 63]. Sex was categorised (male/female) based on genomic analysis and smoking status was self-reported at the Gen2-17 year follow-up (smoked in the past 4 weeks Y/N). The following cell proportion estimates were computed from the DNA methylation data using the Houseman method in the R package *minfi* [64]: CD4T^+^, CD8T^+^, NK, B cells, monocytes, granulocytes. An effect that survived correction for multiple testing was further adjusted for cell proportion estimates, the top five genetic principal components, and the Index of Relative Socio-economic Advantage and Disadvantage (IRSAD) percentile, to investigate potential confounding by cell composition, ancestry, and socio-economic status. The latter measurement, the IRSAD percentile, is a postcode-level measure from the Australian Bureau of Statistics [65].

### Statistical analyses

Analyses were conducted using R version 4.5.1 [66]. All tests are two-sided. Multiple linear regressions were used to test the association between disordered eating and EAA. To test whether disordered eating at age 14 predicted EAA at age 17, separate analyses were performed for each disordered eating measure at age 14 (EDE-Q global scores, restriction, bingeing, purging) and each epigenetic clock (PhenoAge, GrimAge, DunedinPACE, Horvath, Hannum). Two staged models were run for each analysis. First, a basic model was performed to test the relationship between disordered eating at age 14 and EAA at age 17, controlling for sex and disordered eating at age 17. Secondly, a lifestyle model was performed to test the association between age 14 disordered eating and age 17 EAA, adjusting for sex, disordered eating at age 17, age 17 BMI centile, and smoking status.

To investigate whether age 17 disordered eating was associated with age 17 EAA, analyses were repeated using age 17 rather than age 14 disordered eating measures (EDE-Q global scores, restriction, bingeing, purging). The basic models included sex as a covariate, and the lifestyle models controlled for sex, age 17 BMI centile, and smoking status. False discovery rate (FDR) adjusted *p* values (*Q*) were calculated using the Benjamini-Hochberg method to correct for multiple testing [67].

Residual plots were inspected to assess assumptions of normality, linearity, and homoscedasticity. There were modest deviations from normality for some analyses, but – as evidenced by Lumley et al. [68] – the results were likely to be robust to these deviations, given the large sample size. There were no major violations of the linearity assumption. Some scale-location plots showed a slope, evidencing heteroscedasticity for some analyses. Sensitivity analyses were performed for these analyses using heteroscedasticity-consistent standard errors [69]. Significance (at the nominal threshold of 0.05) and the direction of the effect were unchanged, suggesting the results are robust to potential heteroscedasticity. All variance inflation factors were below 5, except in the fully-adjusted model for age 14 restraint and DunedinPACE, where a variance inflation factor above 5 was observed for the cell proportion estimates. As we were not drawing inferences from the estimates for the individual cell proportion estimates, this made no difference to the results – the analysis still controlled for them appropriately.

### Mediation

Serial mediation analysis was used to test whether BMI mediated the relationship between restriction at age 14 and EAA. The mediation analysis was performed with 10,000 bootstrap samples using PROCESS Model 6 version 4.3.1 in R [70]. Sex, smoking status, and age 17 disordered eating were included as covariates.

## Results

### Participant characteristics

Participant characteristics are displayed in Table 1. Participants showed a small significant increase in mean global EDE-Q scores (*t* = 4.08, *p *< 0.001, Cohen’s *d* = 0.14), restriction (*t* = 3.51, *p*  < 0.001, Cohen’s *d* = 0.12), and purging (*t* = 5.72, *p*  < 0.001, Cohen’s *d* = 0.20) between the Gen2-14 year and Gen2-17 year follow-ups, but no significant difference in bingeing (*p* = 0.233).

**Table 1:** Participant Characteristics.

*N* = 797	Age 14	Age 17
Age (*M*, *SD*)	14.08 (0.17)	17.01 (0.22)
Range	12.99 – 14.78	16.28 – 18.32
Female (*n*, %)	395 (49.6)	395 (49.6)
Smoked in past 4 weeks (*n*, %)		121 (15.2%)
BMI (*M*, *SD*)	21.35 (4.21)	23.15 (5.18)
BMI centile (*M*, *SD*)	0.60 (0.28)	0.59 (0.28)
EDE-Q global score (*M*, *SD*)	0.45 (0.48)	0.56 (0.59)
Restraint (*M*, *SD*)	0.72 (0.78)	0.87 (0.92)
Bingeing (*M*, *SD*)	1.26 (1.12)	1.19 (1.22)
Purging (*M*, *SD*)	0.03 (0.18)	0.13 (0.46)
EAA (*M*, *SD*, range)		
PhenoAge^1^		−0.15 (2.93) [−17.71 – 18.07]
GrimAge^1^		−0.20 (5.58) [−9.32 – 12.53]
DunedinPACE^2^		0.86 (0.10) [0.51 – 1.30]
Horvath^1^		−0.12 (3.68) [−14.08 – 11.89]
Hannum_1_		−0.27 (5.33) [−18.15 – 15.60]

Smoking data available for *n* = 717 at age 17. BMI data available for *n* = 796 at age 17. ^1^Negative values indicate slower biological ageing relative to chronological age and positive values indicate faster biological ageing relative to chronological age. ^2^Values less than 1 indicate slower biological ageing relative to chronological age and values greater than 1 indicate faster biological ageing relative to chronological age.

On average, estimates from all five epigenetic clocks indicated that participants demonstrated slower biological ageing relative to chronological age. Correlations between chronological age and epigenetic age were low (*r* < 0.081) for all five clocks, likely due to the low variance in chronological age (all participants were approximately 17 years old) (Supplementary Table 1). Median absolute difference for the four clocks that represent age rather than the pace of ageing ranged from 4.7 (Hannum) to 8.86 (PhenoAge).

### Associations between age 14 disordered eating and age 17 EAA

#### EDE-Q global scores

No significant associations were identified between age 14 EDE-Q global scores and EAA estimates calculated using the PhenoAge, GrimAge, Horvath, or Hannum epigenetic clocks. There was a nominally significant positive association between age 14 EDE-Q global scores and DunedinPACE using the basic covariate model (sex, age 17 EDE-Q global scores) and in the lifestyle model (sex, age 17 EDE-Q global scores, age 17 BMI centile, smoking status) (Table 2), but neither effect survived the FDR correction.

**Table 2:** Regressions Predicting EAA from Age 14 Global EDE-Q Scores.

	*b*	*SE*	*t* value	*p*	Q
PhenoAge					
Basic	0.93	0.52	1.79	0.074	0.37
Lifestyle^1^	0.56	0.56	1.02	0.308	0.678
GrimAge					
Basic	0.20	0.28	0.72	0.470	0.783
Lifestyle^1^	0.20	0.29	0.77	0.441	0.769
DunedinPACE					
Basic	0.03	0.01	3.04	0.002*	0.08
Lifestyle^1^	0.02	0.01	2.36	0.018*	0.24
Horvath					
Basic	0.26	0.35	0.75	0.452	0.769
Lifestyle^1^	0.29	0.37	0.78	0.435	0.769
Hannum					
Basic	0.76	0.50	1.51	0.131	0.417
Lifestyle^1^	0.41	0.54	0.76	0.449	0.769

*b* unstandardised coefficient, *SE* standard error of *b*, *Q* false discovery rate corrected *p* value. Basic model controlled for sex and age 17 global EDE-Q scores. Lifestyle model controlled for sex, age 17 global EDE-Q scores, age 17 BMI centile, and age 17 smoking status.

*significant at *p* < 0.05. ^1^Analyses excluded *n* = 80 participants with missing smoking status data and *n* = 1 participant with missing age 17 BMI centile data (total *n* = 716).

#### Restriction

There were nominally significant positive associations between age 14 restriction and PhenoAge, Horvath, and Hannum, but they did not pass the correction for multiple testing (Table 3). There was a positive association between age 14 restriction and DunedinPACE using the basic covariate model, which remained significant after the FDR correction. The effect was nominally significant in the lifestyle model (i.e., adjusting for sex, age 17 restriction, BMI centile, and smoking status) and remained significant after further adjustment for cell proportion estimates, genetic principal components, and socio-economic status (IRSAD percentile) (*b* = 0.01 [95% CI = 0.003, 0.19], *SE* = 0.004, β = 0.09 [95% CI = 0.03, 0.15], *t* = 2.79, *p* = 0.005).

**Table 3:** Regressions Predicting EAA from Age 14 Restriction.

	*b*	*SE*	*t* value	*p*	Q
PhenoAge					
Basic	0.55	0.27	2.04	0.042*	0.305
Lifestyle^1^	0.31	0.29	1.10	0.273	0.642
GrimAge					
Basic	0.26	0.14	1.84	0.066	0.37
Lifestyle^1^	0.22	0.15	1.49	0.138	0.417
DunedinPACE					
Basic	0.02	0.00	3.83	< 0.001*	0.008*
Lifestyle^1^	0.01	0.01	2.63	0.009*	0.2
Horvath					
Basic	0.38	0.18	2.16	0.031*	0.248
Lifestyle^1^	0.47	0.19	2.45	0.01*	0.2
Hannum					
Basic	0.58	0.26	2.26	0.024*	0.248
Lifestyle^1^	0.41	0.28	1.47	0.143	0.417

*b* unstandardised coefficient, *SE* standard error of *b, Q* false discovery rate corrected *p* value. Basic model controlled for sex and age 17 restriction. Lifestyle model controlled for sex, age 17 restriction, age 17 BMI centile, and age 17 smoking status.

*significant at *p* < 0.05. ^1^Analyses excluded *n* = 80 participants with missing smoking status data and *n* = 1 participant with missing age 17 BMI centile data (total *n* = 716).

Although the effect for DunedinPACE was nominally significant after covariate adjustment, it was attenuated in the lifestyle model relative to the basic model (*b* = 0.02–0.01). Hierarchical regressions demonstrated that age 17 BMI centile (*R*^*2*^_*Change*_ = 0.03, *F*_*Change*_ = 20.05, *p*  < 0.001) contributed to this attenuation, but smoking status did not (*R*^*2*^_*Change*_ = 0.00, *F*_*Change*_ = 0.46, *p* = 0.498). To determine whether this attenuation was unique to age 17 BMI centile, the lifestyle model was repeated using age 14 BMI centile as a covariate in place of age 17 BMI centile. As in the model with age 17 BMI, the association between age 14 restriction and DunedinPACE was nominally significant (*b* = 0.01, *SE* = 0.005, *p* = 0.016). Age 14 BMI centile contributed significantly to the association between age 14 restriction and DunedinPACE (*R*^*2*^_*Change*_ = 0.02, *F*_*Change*_ = 14.74, *p*  < 0.001), approximately equivalent to the contribution of age 17 BMI centile.

#### Bingeing

No significant associations were found between age 14 bingeing and EAA using PhenoAge, GrimAge, DunedinPACE, Horvath, or Hannum, in the basic or lifestyle models (Supplementary Table 2).

#### Purging

Age 14 purging was nominally significantly, positively associated with Horvath in the lifestyle model, but this effect did not pass the FDR correction (Supplementary Table 3). There were no significant associations between age 14 purging and PhenoAge, GrimAge, DunedinPACE, or Hannum.

### Associations between age 17 disordered eating and age 17 EAA

#### EDE-Q global scores

There were positive associations between age 17 global EDE-Q scores and PhenoAge and DunedinPACE in the basic model, but they did not survive the correction for multiple testing (Supplementary Table 4). There were no significant associations between age 17 global EDE-Q scores and GrimAge, Horvath, or Hannum.

#### Restriction

There were no significant associations between age 17 restriction and PhenoAge, GrimAge, DunedinPACE, Horvath, or Hannum, in the basic or lifestyle models (Supplementary Table 5).

#### Bingeing

Age 17 bingeing was not significantly associated with PhenoAge, GrimAge, DunedinPACE, Horvath, or Hannum in the basic or lifestyle models (Supplementary Table 6).

#### Purging

No significant relationships were found between age 17 purging and PhenoAge, GrimAge, DunedinPACE, Horvath, or Hannum in the basic or lifestyle models (Supplementary Table 7).

### Serial mediation analysis

**Fig. 1:**
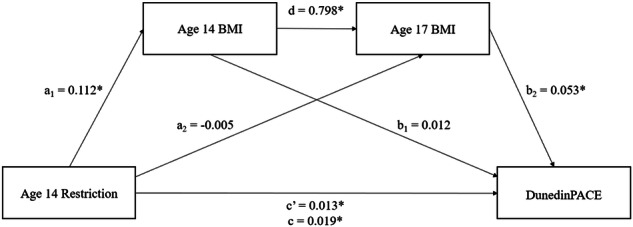
Serial Mediation of Age 14 BMI and Age 17 BMI on the Relationship Between Age 14 Restriction and DunedinPACE Pace of Ageing. Coefficients are unstandardised. a_1_ = effect of age 14 restriction on age 14 BMI centile, a_2_ = effect of age 14 restriction on age 17 BMI centile, b_1_ = effect of age 14 BMI centile on DunedinPACE, b_1_ = effect of age 17 BMI centile on DunedinPACE, d = effect of age 14 BMI centile on age 17 BMI centile, c’ = direct effect of age 14 restriction on DunedinPACE, c = total effect (c’ + ab) of age 14 restriction on DunedinPACE. *Significant at p  <  0.05.

In the regressions testing the relationship between restriction at age 14 and DunedinPACE, the inclusion of BMI and smoking status as covariates in the lifestyle model attenuated the significant association. Hierarchical regressions demonstrated that BMI centile at ages 14 and 17 explained a significant proportion of variance, but smoking status did not. A mediation analysis was therefore used to investigate if BMI mediated the relationship between age 14 restriction and DunedinPACE. Specifically, a serial mediation model tested the indirect effect of age 14 restriction on age 17 pace of ageing through age 14 and age 17 BMI (Fig. 1).

Age 14 restriction had a significant positive effect on age 14 BMI centile (*p* < 0.001) but not age 17 BMI centile (*p* = 0.596). Age 14 BMI centile did not influence DunedinPACE (*p* = 0.591), but age 17 BMI centile had a significant positive impact on DunedinPACE (*p* = 0.020). Age 14 BMI centile had a significant positive effect on age 17 BMI centile (*p* < 0.001), and the total effect of age 14 restriction on DunedinPACE was significant and positive (*b* = 0.02, *SE* = 0.005, *p*  < 0.001). The indirect effect of age 14 restriction on DunedinPACE through age 14 and age 17 BMI centile was significant and positive (*b* = 0.005 [95% CI = 0.001, 0.009], *SE* = 0.002, *p*  < 0.05), with the direct effect of age 14 restriction on DunedinPACE remaining significant in the serial mediation model (*b* = 0.013, *SE* = 0.005, *p* = 0.014), demonstrating a partial mediation of this relationship through BMI. To explore an alternative possible pathway, a separate model was constructed investigating age 14 and age 17 restriction as potential serial mediators between age 14 BMI centile and DunedinPACE. There was no evidence for a significant indirect effect of age 14 BMI on DunedinPACE through restriction at the two time points (*b* = −0.003 [95% CI = −0.01, 0.006], *SE* = 0.005).

## Discussion

The present study was the first to investigate the relationship between disordered eating and measures of epigenetic ageing, and the mediating role of BMI, addressing a substantial gap in the literature. The longitudinal relationship between higher restriction at age 14 and accelerated pace of ageing (DunedinPACE) at age 17 was significant in the basic covariate model, but not in the lifestyle model. Although significant, the effect was small, suggesting restriction at age 14 may only be minimally associated with the pace of ageing. The relationship was partially mediated through BMI at ages 14 and 17, indicating that disordered eating may have contributed to increased BMI, which may have then led to increased pace of ageing. Although whole blood DNA methylation data were used, adjustment for cell proportion estimates indicated no evidence of confounding by cell composition. After the FDR correction, no longitudinal relationships were observable between age 14 bingeing or purging and age 17 EAA, and disordered eating had no short-term effects on EAA.

A key finding was that disordered eating was – after correction for multiple testing – associated with DunedinPACE, but not the other clocks. This may be attributable to the differing methods used to develop each clock. PhenoAge and GrimAge are both second-generation epigenetic clocks that are predictive of mortality [7, 9]. Although mortality rates are elevated among those with eating disorders, particularly anorexia nervosa [71, 72], we used data from the Raine Study, which is representative of the general population. This meant that the number of anorexia nervosa cases was extremely low (*n* = 1), therefore mortality clocks may not have captured elevated mortality risk resulting from disordered eating in this sample. Thus, the impact on mortality may be restricted to more extreme examples of disordered eating (i.e., eating disorders that meet clinical diagnostic criteria) rather than disordered eating and behaviours in the general population.

In contrast to the other epigenetic clocks in this study, DunedinPACE is a third-generation clock that was designed to measure the pace of biological ageing. While the other clocks include biomarkers related to metabolic health and immune response, DunedinPACE measures biomarkers related to various physiological systems, including BMI specifically [73], potentially making it better suited to capturing the broad range of health outcomes associated with disordered eating [1–3, 10]. The other clocks were designed to predict epigenetic age based on cross-sectional data at one timepoint in middle-aged and older adults [7, 9], whereas DunedinPACE was developed from longitudinal data that analysed the decline in 19 biomarkers across two decades (from ages 26 – 45 years) [8, 10], resulting in a measure of the pace an individual is expected to age in a year. This means that DunedinPACE is able to distinguish ageing effects from disease processes and cohort effects, potentially providing a more informative assessment of accelerated ageing compared to clocks that estimate a fixed biological age [10]. Additionally, DunedinPACE may be more sensitive to biological changes within an individual, which may be impacted by disordered eating.

Previous studies investigating EAA across multiple clocks have similarly found DunedinPACE to be a stronger predictor of epigenetic age in phenotypes related to disordered eating. For example, Föhr et al. [74] investigated EAA using DunedinPACE and GrimAge in individuals with metabolic syndrome, which is linked to both disordered eating (particularly binge eating) and BMI [74–77]. While associations with both clocks were significant in basic models (adjusting for family relatedness, age, and sex), only DunedinPACE remained significant in the lifestyle model (additionally adjusting for smoking status, alcohol consumption, and physical activity) [74]. This suggests that DunedinPACE may be more sensitive to biological outcomes related to metabolic syndrome, many of which are shared with binge eating and increased BMI (e.g., increased risk of cardiovascular disease and type 2 diabetes) [78–81]. Studies investigating diet quality have also reported stronger associations with DunedinPACE compared to PhenoAge and GrimAge. Waziry et al. [82] reported that caloric restriction slowed the DunedinPACE pace of ageing, but had no effect on PhenoAge or GrimAge [82]. Although this may seem contradictory to our finding that restriction was associated with an accelerated pace of ageing, Waziry et al. [82] defined caloric restriction as reducing caloric intake whilst maintaining essential nutrient intake. In those with disordered eating, however, restriction typically leads to inadequate nutrient intake [83, 84], indicating that nutrient intake could be crucial in age-related biological changes. Reynolds et al. [85] supports this, reporting that diet quality was most strongly associated with DunedinPACE, with higher diet quality predicting a slower pace of ageing [85]. Finally, Li et al. [86] reported positive cross-sectional associations between body weight and DunedinPACE, GrimAge, and PhenoAge, with strongest associations for DunedinPACE [86]. These findings again support the possibility that DunedinPACE may be more sensitive to biological changes related to disordered eating compared to PhenoAge and GrimAge. Li et al. [86] also reported that both weight gain and weight loss were longitudinally associated with accelerated epigenetic aging, aligning with our analyses demonstrating that increased BMI centile partially mediated the association between disordered eating at age 14 and pace of ageing at age 17 [86].

Our finding that BMI partially mediated the effect of restriction supports previous research linking adolescent disordered eating to increased BMI [87], and research associating higher BMI with increased DNA methylation [25, 28, 37–41]. Although restriction in clinical eating disorder populations often leads to weight loss resulting in very low BMI [4], disordered eating behaviours, including restriction, in the general adolescent population are longitudinally associated with an increase in BMI [87, 88]. As the present sample represents the general adolescent population, our finding that increased BMI centile mediated the relationship between disordered eating and pace of ageing supports existing literature. Specifically, age 14 disordered eating predicted age 14 BMI but not age 17 BMI, while age 14 BMI strongly predicted both age 17 BMI and pace of ageing, suggesting age 14 BMI may play a key role in the increased pace of ageing observed longitudinally. An alternative model with restriction as a mediator between BMI and DunedinPACE revealed no support for an indirect effect, consistent with an effect of restriction on BMI rather than the inverse.

This study has some limitations to consider. Despite using a good sample size (*N* = 797), the prevalence of disordered eating was low, with less than 3% of participants (*n* = 19) at age 14 and less than 5% (*n* = 38) at age 17 scoring above the ‘normal’ EDE-Q score according to Australian community norms (i.e.,  < 1.81) [89]. Because this study did not assess clinically disordered eating specifically, the results may not be generalisable beyond the subclinical phenotype, and further studies are required to determine if the results apply to clinical populations. Also, because the Raine Study sample is representative of the Western Australian population, the results may not be generalisable to other populations. Moreover, while the use of a general population sample increases generalisability and explores the effects of non-clinical disordered eating, we may have been underpowered to detect effects at the epigenetic level if a disordered eating threshold is necessary to detect them. Replication in multiple large cohorts would enable more powerful analyses, and – crucially – capacity to explore if there may be thresholds for disordered eating to have a functional epigenetic impact. Further research may also be warranted in community cohorts specifically, as some of the observed effects for global disordered eating were nominally significant but did not survive the correction for multiple testing. Although disordered eating was assessed using questions adapted from the EDE-Q, which is a validated and widely-used self-report questionnaire [90], global EDE-Q scores were limited to the shortened version administered at age 17, which may not have fully captured the extent of disordered eating symptoms in the sample. Moreover, it is important to note that causality cannot be established, given the design of the current study, which only measured the key variables – disordered eating and EAA – at a single time point each. More targeted studies tracking disordered eating, BMI, and the pace of ageing across multiple time points are required to improve causal inference. Future research may also investigate the possible role of psychiatric comorbidities. For example, there may be interaction effects between disordered eating and personality [91] or substance use disorders [92], whereby the joint effect on EAA is multiplicative rather than additive.

This study revealed a small longitudinal relationship between restrictive disordered eating behaviour at age 14 and an increased pace of ageing at age 17. The association was partially mediated through BMI at ages 14 and 17, consistent with a role for BMI in the adverse health outcomes associated with disordered eating. Findings suggest that third generation clocks may capture some of the long-term biological correlates of disordered eating, but – because the effect was small – it may not be clinically relevant. At the same time, this effect raises the possibility of an epigenetic biomarker specifically tailored to capture the effects of disordered eating – this may be a fruitful area for future research. The findings of the present study are also consistent with the possibility that BMI may play a role in the biological processes associated with disordered eating, highlighting the importance of considering BMI in future treatment approaches for disordered eating. Replication efforts in large community samples and clinical populations are required, along with studies that measure disordered eating and epigenetic age at multiple time points, to improve causal inference.

Supplementary information is available at MP’s website.

## Supplementary information

Supplementary Tables

## Data Availability

Following the Raine Study Researcher Engagement Policy and in support of a high level of participant confidentiality, the data used in the current study are not publicly available. Access can be requested through a formal application process on the Raine Study website.

## References

[1] Hambleton A, Pepin G, Le A, Maloney D, Touyz S, Maguire S. Psychiatric and medical comorbidities of eating disorders: findings from a rapid review of the literature. J Eat Disord. 2022;10:132. 36064606 10.1186/s40337-022-00654-2 PMC9442924

[2] Mitchell JE, Crow S. Medical complications of anorexia nervosa and bulimia nervosa. Curr Opin Psychiatry. 2006;19:438–43. 16721178 10.1097/01.yco.0000228768.79097.3e

[3] Westmoreland P, Krantz MJ, Mehler PS. Medical complications of anorexia nervosa and bulimia. Am J Med. 2016;129:30–7. 26169883 10.1016/j.amjmed.2015.06.031

[4] Diagnostic and statistical manual of mental disorders : DSM-5-TR. 5th edition, text revision. ed. Washington, DC: American Psychiatric Association Publishing; 2022.

[5] Thaler L, Steiger H. Eating disorders and epigenetics. Neuroepigenomics Aging Dis. 2017;978:93–103. 10.1007/978-3-319-53889-1_5 28523542

[6] Guo J, Huang X, Dou L, Yan M, Shen T, Tang W, et al. Aging and aging-related diseases: from molecular mechanisms to interventions and treatments. Signal Transduct Target Ther. 2022;7:391. 36522308 10.1038/s41392-022-01251-0 PMC9755275

[7] Levine ME, Lu AT, Quach A, Chen BH, Assimes TL, Bandinelli S, et al. An epigenetic biomarker of aging for lifespan and healthspan. Aging. 2018;10:573. 29676998 10.18632/aging.101414 PMC5940111

[8] Belsky DW, Caspi A, Houts R, Cohen HJ, Corcoran DL, Danese A, et al. Quantification of biological aging in young adults. Proc Natl Acad Sci. 2015;112:E4104–E10. 26150497 10.1073/pnas.1506264112 PMC4522793

[9] Lu AT, Quach A, Wilson JG, Reiner AP, Aviv A, Raj K, et al. DNA methylation GrimAge strongly predicts lifespan and healthspan. Aging (Albany NY). 2019;11:303. 30669119 10.18632/aging.101684 PMC6366976

[10] Belsky DW, Caspi A, Corcoran DL, Sugden K, Poulton R, Arseneault L, et al. DunedinPACE, a DNA methylation biomarker of the pace of aging. Elife. 2022;11:e73420. 35029144 10.7554/eLife.73420 PMC8853656

[11] Horvath S. DNA methylation age of human tissues and cell types. Genome Biol. 2013;14:1–20. 10.1186/gb-2013-14-10-r115 PMC4015143 24138928

[12] Chuang JC, Jones PA. Epigenetics and microRNAs. Pediatric Res. 2007;61:24–9. 10.1203/pdr.0b013e3180457684 17413852

[13] Dupont C, Armant DR, Brenner CA. Epigenetics: definition, mechanisms and clinical perspective. Semin Reprod Med. 2009;27:351–7. 19711245 10.1055/s-0029-1237423 PMC2791696

[14] Käver L, Hinney A, Rajcsanyi LS, Maier HB, Frieling H, Steiger H, et al. Epigenetic alterations in patients with anorexia nervosa—a systematic review. Mol Psychiatry. 2024;29:1–15. 38849516 10.1038/s41380-024-02601-w PMC11609096

[15] Groleau P, Joober R, Israel M, Zeramdini N, DeGuzman R, Steiger H. Methylation of the dopamine D2 receptor (DRD2) gene promoter in women with a bulimia-spectrum disorder: associations with borderline personality disorder and exposure to childhood abuse. J Psychiatr Res. 2014;48:121–7. 24157248 10.1016/j.jpsychires.2013.10.003

[16] Frieling H, Römer KD, Scholz S, Mittelbach F, Wilhelm J, De Zwaan M, et al. Epigenetic dysregulation of dopaminergic genes in eating disorders. Int J Eat Disord. 2010;43:577–83. 19728374 10.1002/eat.20745

[17] Frieling H, Bleich S, Otten J, Römer KD, Kornhuber J, De Zwaan M, et al. Epigenetic downregulation of atrial natriuretic peptide but not vasopressin mRNA expression in females with eating disorders is related to impulsivity. Neuropsychopharmacology. 2008;33:2605–9. 18172431 10.1038/sj.npp.1301662

[18] Thaler L, Gauvin L, Joober R, Groleau P, de Guzman R, Ambalavanan A, et al. Methylation of BDNF in women with bulimic eating syndromes: associations with childhood abuse and borderline personality disorder. Prog Neuro-psychopharmacol Biol Psychiatry. 2014;54:43–9. 10.1016/j.pnpbp.2014.04.010 24801751

[19] Rodríguez-López ML, Martínez-Magaña JJ, Ruiz-Ramos D, García AR, Gonzalez L, Tovilla-Zarate CA, et al. Individuals diagnosed with binge-eating disorder have DNA hypomethylated sites in genes of the metabolic system: a pilot study. Nutrients. 2021;13:1413. 33922358 10.3390/nu13051413 PMC8145109

[20] Frieling H, Albrecht H, Jedtberg S, Gozner A, Lenz B, Wilhelm J, et al. Elevated cannabinoid 1 receptor mRNA is linked to eating disorder related behavior and attitudes in females with eating disorders. Psychoneuroendocrinology. 2009;34:620–4. 19046818 10.1016/j.psyneuen.2008.10.014

[21] Subramanian S, Braun PR, Han S, Potash JB. Investigation of differential HDAC4 methylation patterns in eating disorders. Psychiatr Genet. 2018;28:12–5. 29256967 10.1097/YPG.0000000000000189 PMC5741469

[22] Steiger H, Labonté B, Groleau P, Turecki G, Israel M. Methylation of the glucocorticoid receptor gene promoter in bulimic women: associations with borderline personality disorder, suicidality, and exposure to childhood abuse. Int J Eat Disord. 2013;46:246–55. 23417893 10.1002/eat.22113

[23] Hübel C, Marzi SJ, Breen G, Bulik CM. Epigenetics in eating disorders: a systematic review. Mol Psychiatry. 2019;24:901–15. 30353170 10.1038/s41380-018-0254-7 PMC6544542

[24] Iranzo-Tatay C, Hervás-Marín D, Rojo-Bofill L, Garcia D, Vaz-Leal F, Calabria I, et al. Genome-wide DNA methylation profiling in anorexia nervosa discordant identical twins. Transl Psychiatry. 2022;12:15. 35013117 10.1038/s41398-021-01776-y PMC8748827

[25] Steiger H, Booij L, Kahan E, McGregor K, Thaler L, Fletcher E, et al. A longitudinal, epigenome-wide study of DNA methylation in anorexia nervosa: results in actively ill, partially weight-restored, long-term remitted and non-eating-disordered women. J Psychiatry Neurosci. 2019;44:205–13. 30693739 10.1503/jpn.170242 PMC6488489

[26] Booij L, Casey KF, Antunes JM, Szyf M, Joober R, Israël M, et al. DNA methylation in individuals with anorexia nervosa and in matched normal‐eater controls: A genome‐wide study. Int J Eat Disord. 2015;48:874–82. 25808061 10.1002/eat.22374

[27] Kesselmeier M, Pütter C, Volckmar A-L, Baurecht H, Grallert H, Illig T, et al. High-throughput DNA methylation analysis in anorexia nervosa confirms TNXB hypermethylation. World J Biol Psychiatry. 2018;19:187–99. 27367046 10.1080/15622975.2016.1190033

[28] Steiger H, Booij L, Thaler L, St-Hilaire A, Israël M, Casey KF, et al. DNA methylation in people with anorexia nervosa: Epigenome-wide patterns in actively ill, long-term remitted, and healthy-eater women. World J Biol Psychiatry. 2023;24:254–9. 35703085 10.1080/15622975.2022.2089731

[29] Anderson OS, Sant KE, Dolinoy DC. Nutrition and epigenetics: an interplay of dietary methyl donors, one-carbon metabolism and DNA methylation. J Nutritional Biochem. 2012;23:853–9. 10.1016/j.jnutbio.2012.03.003 PMC3405985 22749138

[30] Vinkers CH, Kalafateli AL, Rutten BP, Kas MJ, Kaminsky Z, Turner JD, et al. Traumatic stress and human DNA methylation: a critical review. Epigenomics. 2015;7:593–608. 26111031 10.2217/epi.15.11

[31] Harkess K, Ryan J, Delfabbro PH, Cohen-Woods S. Preliminary indications of the effect of a brief yoga intervention on markers of inflammation and DNA methylation in chronically stressed women. Transl Psychiatry. 2016;6:e965. 27898068 10.1038/tp.2016.234 PMC5290356

[32] Landen S, Voisin S, Craig JM, McGee SL, Lamon S, Eynon N. Genetic and epigenetic sex-specific adaptations to endurance exercise. Epigenetics. 2019;14:523–35. 30957644 10.1080/15592294.2019.1603961 PMC6557612

[33] Horvath S, Raj K. DNA methylation-based biomarkers and the epigenetic clock theory of ageing. Nat Rev Genet. 2018;19:371–84. 29643443 10.1038/s41576-018-0004-3

[34] Teschendorff AE, Horvath S. Epigenetic ageing clocks: statistical methods and emerging computational challenges. Nat Rev Genet. 2025;26:350–68. 39806006 10.1038/s41576-024-00807-w

[35] Chervova O, Panteleeva K, Chernysheva E, Widayati TA, Baronik ŽF, Hrbková N, et al. Breaking new ground on human health and well-being with epigenetic clocks: a systematic review and meta-analysis of epigenetic age acceleration associations. Ageing Res Rev. 2024;102:102552. 39423872 10.1016/j.arr.2024.102552

[36] Goldschmidt AB, Aspen VP, Sinton MM, Tanofsky‐Kraff M, Wilfley DE. Disordered eating attitudes and behaviors in overweight youth. Obesity. 2008;16:257–64. 18239631 10.1038/oby.2007.48

[37] Kim Y-R, Kim J-H, Kim MJ, Treasure J. Differential methylation of the oxytocin receptor gene in patients with anorexia nervosa: a pilot study. PLoS one. 2014;9:e88673. 24523928 10.1371/journal.pone.0088673 PMC3921190

[38] Oblak L, van der Zaag J, Higgins-Chen AT, Levine ME, Boks MP. A systematic review of biological, social and environmental factors associated with epigenetic clock acceleration. Ageing Res Rev. 2021;69:101348. 33930583 10.1016/j.arr.2021.101348

[39] Huang R-C, Lillycrop KA, Beilin LJ, Godfrey KM, Anderson D, Mori TA, et al. Epigenetic age acceleration in adolescence associates with BMI, inflammation, and risk score for middle age cardiovascular disease. J Clin Endocrinol Metab. 2019;104:3012–24. 30785999 10.1210/jc.2018-02076 PMC6555851

[40] Lundgren S, Kuitunen S, Pietiläinen KH, Hurme M, Kähönen M, Männistö S, et al. BMI is positively associated with accelerated epigenetic aging in twin pairs discordant for body mass index. J Intern Med. 2022;292:627–40. 35699258 10.1111/joim.13528 PMC9540898

[41] Quach A, Levine ME, Tanaka T, Lu AT, Chen BH, Ferrucci L, et al. Epigenetic clock analysis of diet, exercise, education, and lifestyle factors. Aging. 2017;9:419. 28198702 10.18632/aging.101168 PMC5361673

[42] Huang RC, Melton PE, Burton MA, Beilin LJ, Clarke-Harris R, Cook E, et al. Adiposity associated DNA methylation signatures in adolescents are related to leptin and perinatal factors. Epigenetics. 2022;17:819–36. 33550919 10.1080/15592294.2021.1876297 PMC9423832

[43] Vehmeijer FOL, Küpers LK, Sharp GC, Salas LA, Lent S, Jima DD, et al. DNA methylation and body mass index from birth to adolescence: meta-analyses of epigenome-wide association studies. Genome Med. 2020;12:105. 33239103 10.1186/s13073-020-00810-w PMC7687793

[44] Straker L, Mountain J, Jacques A, White S, Smith A, Landau L, et al. Cohort profile: the Western Australian pregnancy cohort (Raine) study–generation 2. Int J Epidemiol. 2017;46:1384–5j. 28064197 10.1093/ije/dyw308 PMC5837608

[45] Robinson L, Zhang Z, Jia T, Bobou M, Roach A, Campbell I, et al. Association of genetic and phenotypic assessments with onset of disordered eating behaviors and comorbid mental health problems among adolescents. JAMA Netw Open. 2020;3:e2026874. 33263759 10.1001/jamanetworkopen.2020.26874 PMC7711322

[46] Miller SA, Dykes DD, Polesky H. A simple salting out procedure for extracting DNA from human nucleated cells. Nucleic Acids Res. 1988;16:1215. 3344216 10.1093/nar/16.3.1215 PMC334765

[47] Fortin J-P, Fertig E, Hansen K. shinyMethyl: interactive quality control of Illumina 450k DNA methylation arrays in R. F1000Research. 2014;3:175. 25285208 10.12688/f1000research.4680.1 PMC4176427

[48] Van Iterson M, Tobi EW, Slieker RC, Den Hollander W, Luijk R, Slagboom PE, et al. MethylAid: visual and interactive quality control of large Illumina 450k datasets. Bioinformatics. 2014;30:3435–7. 25147358 10.1093/bioinformatics/btu566

[49] Assenov Y, Müller F, Lutsik P, Walter J, Lengauer T, Bock C. Comprehensive analysis of DNA methylation data with RnBeads. Nat Methods. 2014;11:1138–40. 25262207 10.1038/nmeth.3115 PMC4216143

[50] Bertram CE, Hanson MA. Animal models and programming of the metabolic syndrome: Type 2 diabetes. Br Med Bull. 2001;60:103–21. 11809621 10.1093/bmb/60.1.103

[51] Catalano PM, Kirwan JP. Maternal factors that determine neonatal size and body fat. Curr Diabetes Rep. 2001;1:71–7. 10.1007/s11892-001-0013-y 12762960

[52] Rzehak P, Covic M, Saffery R, Reischl E, Wahl S, Grote V, et al. DNA-methylation and body composition in preschool children: epigenome-wide-analysis in the European Childhood Obesity Project (CHOP)-Study. Sci Rep. 2017;7:14349. 29084944 10.1038/s41598-017-13099-4 PMC5662763

[53] Godfrey KM, Sheppard A, Gluckman PD, Lillycrop KA, Burdge GC, McLean C, et al. Epigenetic gene promoter methylation at birth is associated with child’s later adiposity. Diabetes. 2011;60:1528–34. 21471513 10.2337/db10-0979 PMC3115550

[54] Lillycrop K, Murray R, Cheong C, Teh AL, Clarke-Harris R, Barton S, et al. ANRIL promoter DNA methylation: a perinatal marker for later adiposity. EBioMedicine. 2017;19:60–72. 28473239 10.1016/j.ebiom.2017.03.037 PMC5440605

[55] Teschendorff AE, Marabita F, Lechner M, Bartlett T, Tegner J, Gomez-Cabrero D, et al. A beta-mixture quantile normalization method for correcting probe design bias in Illumina Infinium 450 k DNA methylation data. Bioinformatics. 2013;29:189–96. 23175756 10.1093/bioinformatics/bts680 PMC3546795

[56] Hannum G, Guinney J, Zhao L, Zhang L, Hughes G, Sadda S, et al. Genome-wide methylation profiles reveal quantitative views of human aging rates. Mol Cell. 2013;49:359–67. 23177740 10.1016/j.molcel.2012.10.016 PMC3780611

[57] Fairburn CG, Beglin SJ. Assessment of eating disorders: Interview or self‐report questionnaire? Int J Eat Disord. 1994;16:363–70. 7866415

[58] Bryant-Waugh RJ, Cooper PJ, Taylor CL, Lask BD. The use of the eating disorder examination with children: a pilot study. Int J Eat Disord. 1996;19:391–7. 8859397 10.1002/(SICI)1098-108X(199605)19:4<391::AID-EAT6>3.0.CO;2-G

[59] Allen KL, Byrne SM, Oddy WH, Crosby RD. DSM-IV-TR and DSM-5 eating disorders in adolescents: prevalence, stability, and psychosocial correlates in a population-based sample of male and female adolescents. J Abnorm Psychol. 2013;122:720–32. 24016012 10.1037/a0034004

[60] Berg KC, Peterson CB, Frazier P, Crow SJ. Psychometric evaluation of the eating disorder examination and eating disorder examination-questionnaire: A systematic review of the literature. Int J Eat Disord. 2012;45:428–38. 21744375 10.1002/eat.20931 PMC3668855

[61] Joehanes R, Just AC, Marioni RE, Pilling LC, Reynolds LM, Mandaviya PR, et al. Epigenetic signatures of cigarette smoking. Circulation: Cardiovascular Genet. 2016;9:436–47. 10.1161/CIRCGENETICS.116.001506 PMC5267325 27651444

[62] Kuczmarski RJ 2000 CDC Growth Charts for the United States: methods and development: Department of Health and Human Services, Centers for Disease Control and Prevention, National Center for Health Statistics; 2002.

[63] Vogel M hildsds: Data and Methods Around Reference Values in Pediatrics: R package version 0.8.0; 2022 https://CRAN.R-project.org/package=childsds.

[64] Aryee MJ, Jaffe AE, Corrada-Bravo H, Ladd-Acosta C, Feinberg AP, Hansen KD, et al. Minfi: a flexible and comprehensive Bioconductor package for the analysis of Infinium DNA methylation microarrays. Bioinformatics. 2014;30:1363–9. 24478339 10.1093/bioinformatics/btu049 PMC4016708

[65] Australian Bureau of Statistics. Socio-Economic Indexes for Areas (SEIFA), Australia 2023 https://www.abs.gov.au/statistics/people/people-and-communities/socio-economic-indexes-areas-seifa-australia/latest-release.

[66] Team RC. R: A language and environment for statistical computing Vienna, Austria. R Foundation for Statistical Computing; 2024 https://www.R-project.org/.

[67] Benjamini Y, Hochberg Y. Controlling the false discovery rate: a practical and powerful approach to multiple testing. J R Stat Society: Ser B. 1995;57:289–300.

[68] Lumley T, Diehr P, Emerson S, Chen L. The importance of the normality assumption in large public health data sets. Annu Rev Public Health. 2002;23:151–69. 11910059 10.1146/annurev.publhealth.23.100901.140546

[69] Hayes AF, Cai L. Using heteroskedasticity-consistent standard error estimators in OLS regression: An introduction and software implementation. Behav Res Methods. 2007;39:709–22. 18183883 10.3758/bf03192961

[70] Hayes AF Introduction to mediation, moderation, and conditional process analysis: A regression-based approach: Guilford publications; 2017.

[71] Arcelus J, Mitchell AJ, Wales J, Nielsen S. Mortality rates in patients with anorexia nervosa and other eating disorders: a meta-analysis of 36 studies. Arch Gen Psychiatry. 2011;68:724–31. 21727255 10.1001/archgenpsychiatry.2011.74

[72] Chesney E, Goodwin GM, Fazel S. Risks of all‐cause and suicide mortality in mental disorders: a meta‐review. World Psychiatry. 2014;13:153–60. 24890068 10.1002/wps.20128 PMC4102288

[73] Belsky DW, Caspi A, Arseneault L, Baccarelli A, Corcoran DL, Gao L, et al. Quantification of the pace of biological aging in humans through a blood test, the DunedinPoAm DNA methylation algorithm. eLife. 2020;9:e54870. 32367804 10.7554/eLife.54870 PMC7282814

[74] Föhr T, Hendrix A, Kankaanpää A, Laakkonen EK, Kujala U, Pietiläinen KH, et al. Metabolic syndrome and epigenetic aging: a twin study. Int J Obes. 2024;48:1–10. 10.1038/s41366-024-01466-x PMC11129944 38273034

[75] Hudson JI, Lalonde JK, Coit CE, Tsuang MT, McElroy SL, Crow SJ, et al. Longitudinal study of the diagnosis of components of the metabolic syndrome in individuals with binge-eating disorder. Am J Clin Nutr. 2010;91:1568–73. 20427731 10.3945/ajcn.2010.29203 PMC2869508

[76] Morrison FG, Logue MW, Guetta R, Maniates H, Stone A, Schichman SA, et al. Investigation of bidirectional longitudinal associations between advanced epigenetic age and peripheral biomarkers of inflammation and metabolic syndrome. Aging. 2019;11:3487. 31173577 10.18632/aging.101992 PMC6594822

[77] Roehrig M, Masheb RM, White MA, Grilo CM. The metabolic syndrome and behavioral correlates in obese patients with binge eating disorder. Obesity. 2009;17:481–6. 19219063 10.1038/oby.2008.560 PMC2704920

[78] Eckel RH, Grundy SM, Zimmet PZ. The metabolic syndrome. Lancet. 2005;365:1415–28. 15836891 10.1016/S0140-6736(05)66378-7

[79] Nicolau J, Simó R, Sanchís P, Ayala L, Fortuny R, Zubillaga I, et al. Eating disorders are frequent among type 2 diabetic patients and are associated with worse metabolic and psychological outcomes: results from a cross-sectional study in primary and secondary care settings. Acta Diabetologica. 2015;52:1037–44. 25841588 10.1007/s00592-015-0742-z

[80] Raevuori A, Suokas J, Haukka J, Gissler M, Linna M, Grainger M, et al. Highly increased risk of type 2 diabetes in patients with binge eating disorder and bulimia nervosa. Int J Eat Disord. 2015;48:555–62. 25060427 10.1002/eat.22334

[81] Udo T, McKee SA, White MA, Masheb RM, Barnes RD, Grilo CM. Menopause and metabolic syndrome in obese individuals with binge eating disorder. Eat Behav. 2014;15:182–5. 24854801 10.1016/j.eatbeh.2014.01.003 PMC4032475

[82] Waziry R, Ryan C, Corcoran D, Huffman K, Kobor M, Kothari M, et al. Effect of long-term caloric restriction on DNA methylation measures of biological aging in healthy adults from the CALERIE trial. Nat Aging. 2023;3:248–57. 37118425 10.1038/s43587-022-00357-y PMC10148951

[83] Setnick J. Micronutrient deficiencies and supplementation in anorexia and bulimia nervosa: a review of literature. Nutr Clin Pract. 2010;25:137–42. 20413694 10.1177/0884533610361478

[84] Hanachi M, Dicembre M, Rives-Lange C, Ropers J, Bemer P, Zazzo J-F, et al. Micronutrients deficiencies in 374 severely malnourished anorexia nervosa inpatients. Nutrients. 2019;11:792. 30959831 10.3390/nu11040792 PMC6520973

[85] Reynolds LM, Houston DK, Skiba MB, Whitsel EA, Stewart JD, Li Y, et al. Diet quality and epigenetic aging in the women’s health initiative. J Acad Nutr Dietetics. 2024;124:1419–1430.e3. 10.1016/j.jand.2024.01.002 PMC11236955 38215906

[86] Li DL, Hodge AM, Cribb L, Southey MC, Giles GG, Milne RL, et al. Body size, diet quality, and epigenetic ageing: cross-sectional and longitudinal analyses. J Gerontology, Ser A: Biol Sci Med Sci. 2024;79:glae026. 10.1093/gerona/glae026 PMC10953795 38267386

[87] Neumark-Sztainer D, Wall M, Guo J, Story M, Haines J, Eisenberg M. Obesity, disordered eating, and eating disorders in a longitudinal study of adolescents: how do dieters fare 5 years later? J Am Dietetic Assoc. 2006;106:559–68. 10.1016/j.jada.2006.01.003 16567152

[88] Field AE, Austin SB, Taylor CB, Malspeis S, Rosner B, Rockett HR, et al. Relation between dieting and weight change among preadolescents and adolescents. Pediatrics. 2003;112:900–6. 14523184 10.1542/peds.112.4.900

[89] Mond JM, Hay PJ, Rodgers B, Owen C, Beumont PJ. Validity of the eating disorder examination questionnaire (EDE-Q) in screening for eating disorders in community samples. Behav Res Ther. 2004;42:551–67. 15033501 10.1016/S0005-7967(03)00161-X

[90] Fairburn CG, Beglin SJ. Eating disorder examination questionnaire. Cognit Behav Ther Eat Disord. 2008;309:313.

[91] Martinussen M, Friborg O, Schmierer P, Kaiser S, Øvergård KT, Neunhoeffer L, et al. The comorbidity of personality disorders in eating disorders: a meta-analysis. Eat Weight Disord. 2016;22:201–9. 27995489 10.1007/s40519-016-0345-x

[92] Bahji A, Mazhar MN, Hudson CC, Nadkarni P, MacNeil BA, Hawken E. Prevalence of substance use disorder comorbidity among individuals with eating disorders: a systematic review and meta-analysis. Psychiatry Res. 2019;273:58–66. 30640052 10.1016/j.psychres.2019.01.007

