# Supplementary material for: Adolescent disordered eating and epigenetic age acceleration
Source: Mol Psychiatry. 2026 Apr 10;31(9):5062–9. doi: 10.1038/s41380-026-03595-3 (PMC13441869; doi:10.1038/s41380-026-03595-3)
Supplement: Supplementary file 1 [file 41380_2026_3595_MOESM1_ESM.docx]

**Supplementary Tables**

**Supplementary Table 1**

*Correlation Between Chronological Age and Epigenetic Age.*

|  | CA | PhenoAge | GrimAge | Horvath | Hannum | PACE |
| --- | --- | --- | --- | --- | --- | --- |
| CA | – | 0.053 (.136) | 0.081 (.023) | 0.025 (.489) | 0.054 (0.124) | 0.018 (.602) |
| PhenoAge | 0.053 (.136) | – | 0.454 (<.001) | 0.423 (<.001) | 0.663 (<0.001) | 0.535 (<.001) |
| GrimAge | 0.081 (.023) | 0.454 (<.001) | – | 0.182 (<.001) | 0.489 (<0.001) | 0.495 (<.001) |
| Horvath | 0.025 (.489) | 0.423 (<.001) | 0.182 (<.001) | – | 0.431 (<0.001) | 0.095 (.007) |
| Hannum | 0.054 (0.124) | 0.663 (<0.001) | 0.489 (<0.001) | 0.431 (<0.001) | – | 0.443 (<0.001) |
| PACE | 0.018 (.602) | 0.535 (<.001) | 0.495 (<.001) | 0.095 (.007) | 0.443 (<0.001) | – |

*Note*. Pearson’s *r* with *p* in brackets. CA = chronological age, PACE = DunedinPACE.

**Supplementary Table 2**

*Regressions Predicting EAA from Age 14 Bingeing.*

|  | *b* | *SE* | *t* value | *p* | Q |
| --- | --- | --- | --- | --- | --- |
| PhenoAge |  |  |  |  |  |
| Basic | -0.03 | 0.19 | -0.15 | .879 | .997 |
| Lifestyle^1^ | -0.06 | 0.19 | -0.32 | .753 | .997 |
| GrimAge |  |  |  |  |  |
| Basic | -0.01 | 0.10 | -0.07 | .947 | .997 |
| Lifestyle^1^ | 0.03 | 0.10 | 0.26 | .794 | .997 |
| DunedinPACE |  |  |  |  |  |
| Basic | 0.00 | 0.00 | 1.47 | .142 | .417 |
| Lifestyle^1^ | 0.00 | 0.00 | 1.18 | .238 | .577 |
| Horvath |  |  |  |  |  |
| Basic | -0.002 | 0.12 | -0.02 | .984 | .997 |
| Lifestyle^1^ | 0.018 | 0.13 | 0.14 | .889 | .997 |
| Hannum |  |  |  |  |  |
| Basic | 0.08 | 0.18 | 0.47 | .641 | .916 |
| Lifestyle^1^ | 0.13 | 0.19 | 0.68 | .496 | .794 |

*Note. b =* unstandardised coefficient, *SE* = standard error of *b*, *Q* = false discovery rate corrected *p* value. Basic model controlled for sex and age 17 bingeing scores. Lifestyle model controlled for sex, age 17 bingeing scores scores, age 17 BMI centile, and age 17 smoking status. ^1^Analyses excluded *n =* 80 participants with missing smoking status data and *n* = 1 participant with missing age 17 BMI centile data (total *n* = 716).

**Supplementary Table 3**

*Regressions Predicting EAA from Age 14 Purging.*

|  | *b* | *SE* | *t* value | *p* | Q |
| --- | --- | --- | --- | --- | --- |
| PhenoAge |  |  |  |  |  |
| Basic | 0.62 | 1.10 | 0.56 | .575 | .836 |
| Lifestyle^1^ | 0.71 | 1.13 | 0.63 | .526 | .809 |
| GrimAge |  |  |  |  |  |
| Basic | 0.06 | 0.58 | 0.10 | .922 | .997 |
| Lifestyle^1^ | 0.13 | 0.59 | 0.23 | .822 | .997 |
| DunedinPACE |  |  |  |  |  |
| Basic | 0.03 | 0.02 | 1.74 | .082 | .386 |
| Lifestyle^1^ | 0.03 | 0.02 | 1.67 | .096 | 0.4 |
| Horvath |  |  |  |  |  |
| Basic | 1.3 | 0.72 | 1.79 | .073 | .37 |
| Lifestyle^1^ | 1.66 | 0.76 | 2.2 | .028* | .248 |
| Hannum |  |  |  |  |  |
| Basic | -0.008 | 1.05 | -0.008 | .994 | .997 |
| Lifestyle^1^ | -0.12 | 1.08 | -0.11 | .915 | .997 |

*Note. b =* unstandardised coefficient, *SE* = standard error of *b*, *Q* = false discovery rate corrected *p* value. Basic model controlled for sex and age 17 purging scores. Lifestyle model controlled for sex, age 17 purging scores scores, age 17 BMI centile, and age 17 smoking status. *significant at *p* < .05. ^1^Analyses excluded *n =* 80 participants with missing smoking status data and *n* = 1 participant with missing age 17 BMI centile data (total *n* = 716).

**Supplementary Table 4**

*Regressions Predicting EAA from Age 17 Global EDE-Q Scores.*

|  | *b* | *SE* | *t* value | *P* | Q |
| --- | --- | --- | --- | --- | --- |
| PhenoAge |  |  |  |  |  |
| Basic | 0.81 | 0.37 | 2.17 | .031* | .248 |
| Lifestyle^1^ | -0.04 | 0.42 | -0.09 | .928 | .997 |
| GrimAge |  |  |  |  |  |
| Basic | 0.35 | 0.20 | 1.80 | .073 | .37 |
| Lifestyle^1^ | 0.28 | 0.22 | 1.26 | .207 | .552 |
| DunedinPACE |  |  |  |  |  |
| Basic | 0.01 | 0.01 | 2.39 | .017* | .24 |
| Lifestyle^1^ | 0.0008 | 0.01 | 0.11 | .914 | .997 |
| Horvath |  |  |  |  |  |
| Basic | 0.19 | 0.25 | 0.77 | .439 | .769 |
| Lifestyle^1^ | -0.001 | 0.28 | -0.03 | .997 | .997 |
| Hannum |  |  |  |  |  |
| Basic | 0.30 | 0.36 | 0.84 | .4 | .769 |
| Lifestyle^1^ | -0.03 | 0.41 | -0.08 | .94 | .997 |

*Note. b =* unstandardised coefficient, *SE* = standard error of *b, Q* = false discovery rate corrected *p* value. Basic model controlled for sex. Lifestyle model controlled for sex, age 17 BMI centile, and age 17 smoking status. *significant at *p* < .05. ^1^Analyses excluded *n =* 80 participants with missing smoking status data (total *n* = 717).

**Supplementary Table 5**

*Regressions Predicting EAA from Age 17 Restriction.*

|  | *b* | *SE* | *t* value | *p* | Q |
| --- | --- | --- | --- | --- | --- |
| PhenoAge |  |  |  |  |  |
| Basic | 0.36 | 0.21 | 1.67 | .096 | .4 |
| Lifestyle^1^ | -0.01 | 0.24 | -0.03 | .978 | .997 |
| GrimAge |  |  |  |  |  |
| Basic | 0.18 | 0.11 | 1.58 | .114 | .415 |
| Lifestyle^1^ | 0.20 | 0.12 | 1.61 | .107 | .408 |
| DunedinPACE |  |  |  |  |  |
| Basic | 0.01 | 0.00 | 1.49 | .138 | .417 |
| Lifestyle^1^ | 0.00 | 0.00 | -0.07 | .945 | .997 |
| Horvath |  |  |  |  |  |
| Basic | 0.11 | 0.14 | 0.78 | .439 | .769 |
| Lifestyle^1^ | 0.03 | 0.16 | 0.18 | .859 | .997 |
| Hannum |  |  |  |  |  |
| Basic | 0.02 | 0.21 | 0.10 | .920 | .997 |
| Lifestyle^1^ | -0.16 | 0.23 | -0.69 | .489 | .794 |

*Note. b =* unstandardised coefficient, *SE* = standard error of *b, Q* = false discovery rate corrected *p* value. Basic model controlled for sex. Lifestyle model controlled for sex, age 17 BMI centile, and age 17 smoking status. *significant at *p* < .05. ^1^Analyses excluded *n =* 80 participants with missing smoking status data (total *n* = 717).

**Supplementary Table 6**

*Regressions Predicting EAA from Age 17 Bingeing.*

|  | *b* | *SE* | *t* value | *p* | Q |
| --- | --- | --- | --- | --- | --- |
| PhenoAge |  |  |  |  |  |
| Basic | 0.13 | 0.16 | 0.78 | .435 | .769 |
| Lifestyle^1^ | -0.04 | 0.17 | -0.24 | .814 | .997 |
| GrimAge |  |  |  |  |  |
| Basic | 0.08 | 0.09 | 0.96 | .339 | .695 |
| Lifestyle^1^ | 0.06 | 0.09 | 0.65 | .519 | .809 |
| DunedinPACE |  |  |  |  |  |
| Basic | 0.00 | 0.00 | 0.27 | .789 | .997 |
| Lifestyle^1^ | 0.00 | 0.00 | -0.99 | .322 | .678 |
| Horvath |  |  |  |  |  |
| Basic | 0.01 | 0.11 | 0.12 | .903 | .997 |
| Lifestyle^1^ | -0.04 | 0.12 | -0.32 | .750 | .997 |
| Hannum |  |  |  |  |  |
| Basic | 0.02 | 0.16 | 0.11 | .91 | .997 |
| Lifestyle^1^ | -0.04 | 0.16 | -0.27 | .786 | .997 |

*Note. b =* unstandardised coefficient, *SE* = standard error of *b, Q* = false discovery rate corrected *p* value. Basic model controlled for sex. Lifestyle model controlled for sex, age 17 BMI centile, and age 17 smoking status. *significant at *p* < .05. ^1^Analyses excluded *n =* 80 participants with missing smoking status data (total *n* = 717).

**Supplementary Table 7**

*Regressions Predicting EAA from Age 17 Purging.*

|  | *b* | *SE* | *t* value | *p* | Q |
| --- | --- | --- | --- | --- | --- |
| PhenoAge |  |  |  |  |  |
| Basic | 0.71 | 0.43 | 1.65 | .100 | .4 |
| Lifestyle^1^ | 0.53 | 0.43 | 1.22 | .221 | .553 |
| GrimAge |  |  |  |  |  |
| Basic | 0.28 | 0.23 | 1.24 | .215 | .553 |
| Lifestyle^1^ | 0.30 | 0.22 | 1.31 | .190 | .524 |
| DunedinPACE |  |  |  |  |  |
| Basic | 0.01 | 0.01 | 1.46 | .146 | .417 |
| Lifestyle^1^ | 0.01 | 0.01 | 1.00 | .317 | .678 |
| Horvath |  |  |  |  |  |
| Basic | 0.29 | 0.29 | 1.01 | .313 | .678 |
| Lifestyle^1^ | 0.18 | 0.29 | 0.60 | .546 | .824 |
| Hannum |  |  |  |  |  |
| Basic | 0.16 | 0.29 | 0.56 | .575 | .836 |
| Lifestyle^1^ | 0.82 | 0.42 | 1.96 | .0504 | .336 |

*Note. b =* unstandardised coefficient, *SE* = standard error of *b, Q* = false discovery rate corrected *p* value. Basic model controlled for sex. Lifestyle model controlled for sex, age 17 BMI centile, and age 17 smoking status. *significant at *p* < .05. ^1^Analyses excluded *n =* 80 participants with missing smoking status data (total *n* = 717).
